# Overexpression of INSM1, NOTCH1, NEUROD1, and YAP1 genes is associated with adverse clinical outcome in pediatric neuroblastoma

**DOI:** 10.1007/s00428-022-03406-4

**Published:** 2022-09-19

**Authors:** Jasna Metovic, Francesca Napoli, Simona Osella-Abate, Luca Bertero, Cristian Tampieri, Giulia Orlando, Maurizio Bianchi, Diana Carli, Franca Fagioli, Marco Volante, Mauro Papotti

**Affiliations:** 1 Department of Oncology, University of Turin, Orbassano, Turin, Italy; 2 Pathology Unit, “Città della Salute e della Scienza” Hospital, Turin, Italy; 3 Department of Medical Sciences, University of Turin, Turin, Italy; 4 Pediatric Onco-hemathology Unit, “Città della Salute e della Scienza” Hospital, Turin, Italy; 5 Department of Sciences of Public Health and Pediatrics, University of Turin, Turin, Italy

**Keywords:** Neuroblastoma, Gene expression profile, *INSM1*, *NOTCH1*, *NEUROD1*, *YAP1*, Outcome

## Abstract

Pediatric neuroblastoma is responsible for approximately 8–10% of pediatric tumors, and it is one of the leading causes of tumor-related deaths in children. Although significant progress has been made in the characterization of neuroblastoma in recent years, the mechanisms influencing the prognosis of neuroblastoma patients remain largely unknown. Our aim was to investigate if the major neuroendocrine-associated transcriptional drivers, including *ASCL1*, *NEUROD1*, *DLL3*, *NOTCH1*, *INSM1*, *MYCL1*, *POU2F3* and *YAP1* are correlated with specific clinical and pathological characteristics. We selected a retrospective series of 46 primary pediatric neuroblastoma, composed of 30 treatment-naïve and 16 post-chemotherapy cases. Gene expression levels were explored by means of quantitative real-time PCR. An increased expression of *NOTCH1* (*p* = 0.005), *NEUROD1* (*p* = 0.0059), and *YAP1* (*p* = 0.0008) was found in stage IV tumors, while the highest levels of *MYCL1* and *ASCL1* were seen in stages IVS and III, respectively (*p* = 0.0182 and *p* = 0.0134). A higher level of *NOTCH1* (*p* = 0.0079) and *YAP1* (*p* = 0.0026) was found in cases with differentiating morphology, while high mitosis-karyorrhexis index cases demonstrated significantly lower levels of *POU2F3* (*p* = 0.0277). High expression of *NOTCH1* (*p* = 0.008), *NEUROD1* (*p* = 0.026), *INSM1* (*p* = 0.010), and *YAP1* (*p* = 0.005) together with stage IV (*p* = 0.043) was associated with shorter disease-free survival. In summary, our data indicate that the assessment of gene expression levels of neuroendocrine-lineage transcription factors might help to identify neuroblastoma patients with the risk of relapse.

**Supplementary Information:**

The online version contains supplementary material available at 10.1007/s00428-022-03406-4.

## Introduction

Neuroblastic tumors represent the most common extra-cranial solid malignancy in the pediatric age. Neuroblastoma is a tumor arising from developing sympathetic nervous system and is responsible for approximately 8–10% of pediatric tumors. Despite advances in molecular profiling and therapeutic options, survival of high-risk neuroblastoma patients remains poor. Neuroblastoma development mechanisms are incompletely understood, but linked to oncogene mutations and/or amplifications [1–4]. In particular, *MYCN* represents one of the most important driver genes in neuroblastoma, being gene amplification strongly correlated to unfavorable outcome, although in vitro and in vivo data suggest that there is no direct correlation between a high cellular MYCN protein content and aggressive tumor cell behavior or loss of differentiation [5]. Moreover, basic helix-loop-helix (bHLH) class of transcription factors plays a pivotal role in tissue-specific differentiation and their dysregulation is associated to solid tumor development. Neuronal and neuroendocrine (NE) cell growth and differentiation, as well as their related tumors, are regulated by genes of the Notch, NEUROD and Achaete Scute families [6–8]. In particular, in high-grade NE small cell carcinomas, mostly of the lung, a complex genetic regulation has been recently reported with several pathways differentially activated in subgroups of such tumors. Among relevant regulators of NE growth and differentiation are Notch family genes, *ASCL1*, *NEUROD1*, *HES1*, *INSM1*, *POU2F3*, *YAP1*, *MYCL1* [9–12]. In large cell NE carcinomas of the lung, two different molecular profiles were also identified, partly overlapping with those of small cell lung cancer or sharing gene alterations seen in non-small lung carcinomas [13–16]. Some of these genetic differences were linked to different outcome and response to specific therapies in both types of pulmonary high-grade NE carcinomas [10, 17]. In addition, some of these transcription factors are also express in extra-pulmonary NE carcinomas of various locations and are used as markers of NE differentiation [18–20].

Some genes belonging to the abovementioned families have been individually investigated in neuroblastoma and found to be overexpressed and involved in response to specific therapies*.* In particular *MYCN* gene is pivotal in neuroblastoma development and progression [1–4]. Central (intracranial) and peripheral neuroectodermal tumors and cell lines are known to express *NEUROD1* and *ASCL1* genes [21]. Transcriptional regulators of these two genes were recently identified in neuroblastoma, including the neuronal differentiation markers of Purkinje cells *PCP4/PEP19* [22], and *ERK* [23]. *NEUROD1* seems to act mainly through *ALK* to favor neuroblastoma cell proliferation, directly binding to the promoter region of this gene [24]. *ASCL1* is downregulated during neuroblastoma cell differentiation along with upregulation of several genes including *IGF2* [25]; moreover, the negative correlation of *ASCL1* expression with neuronal differentiation is independent from *MYCN* gene expression, suggesting that targeting *ASCL1* might increase the efficacy of retinoic acid-based differentiating therapies in neuroblastoma [26]. More recently, other regulatory genes were investigated, including *INSM1* that is activated by *MYCN* gene, expressed in a large fraction of neuroblastomas and associated to a shorter survival [27–30].

Neuroblastic tumors encompass a spectrum of lesions with different pathological features, response to therapies and outcome. We studied the role of major drivers of neuroendocrine-associated transcriptional clustering in a retrospective series of surgically resected pediatric neuroblastomas, with the aim of exploring the correlation with specific pathological and clinical characteristics including response to treatment and survival.

## Materials and methods

### Case series

We selected a retrospective series of 46 cases of pediatric neuroblastoma, all primitive, operated from 2007 to 2019 at “Città della Salute e della Scienza,” Turin, and treated at the Pediatric Oncology Division of the “Regina Margherita” Children’s Hospital, with sufficient residual histological material for molecular and immunohistochemical analyses. The case series was composed 30 treatment-naïve and 16 post-chemotherapy cases. The chemotherapy consisted of combination treatment, including carboplatin and etoposide.

Clinical and pathological data such as age at diagnosis, sex, tumor location, stage (according to INSS system, see Table 1), International Neuroblastoma Pathology classification (INPC) category [31], mitosis-karyorrhexis index (MKI) according to Shimada classification [32], presence of necrosis, calcifications, and follow-up data were collected from clinical charts and inserted in a dedicated database.

**Table 1:** International Neuroblastoma Staging System (INSS)

Stage	Description
1	The cancer is still in the area where it started. It is on one side of the body (right or left). All visible tumor has been removed completely by surgery (although looking at the tumor’s edges under the microscope after surgery may show some cancer cells). Lymph nodes near the tumor are free of cancer (although nodes enclosed within the tumor may contain neuroblastoma cells)
2A	The cancer is still in the area where it started and on one side of the body, but not all of the visible tumor could be removed by surgery. Lymph nodes near the tumor are free of cancer (although nodes enclosed within the tumor may contain neuroblastoma cells)
2B	The cancer is on one side of the body, and it may or may not have been removed completely by surgery. Nearby lymph nodes outside the tumor contain neuroblastoma cells, but the cancer has not spread to lymph nodes on the other side of the body or elsewhere
3	The cancer has not spread to distant parts of the body, but one of the following is true: a) The cancer can't be removed completely by surgery, and it has crossed the midline (defined as the spine) to the other side of the body. It may or may not have spread to nearby lymph nodes b) The cancer is still in the area where it started and is on one side of the body. It has spread to lymph nodes that are relatively nearby but on the other side of the body c) The cancer is in the middle of the body and is growing toward both sides (either directly or by spreading to nearby lymph nodes)
4	The cancer has spread to distant parts of the body such as distant lymph nodes, bones, liver, skin, bone marrow, or other organs (but the child does not meet the criteria for stage 4S)
4S	The child is younger than 1 year old. The cancer is on one side of the body. It might have spread to lymph nodes on the same side of the body but not to nodes on the other side. The neuroblastoma has spread to the liver, skin, and/or the bone marrow. However, no more than 10% of marrow cells are cancer cells, and imaging tests such as an MIBG scan do not show cancer in the bone marrow

(from: https://www.cancer.org/cancer/neuroblastoma/detection-diagnosis-staging/staging.html)

### RNA extraction from formalin-fixed paraffin-embedded tissues and gene expression analyses

Ten µm thick sections were cut were cut from formalin fixed paraffin embedded blocks of the tumor in RNase-free conditions, following microdissection using a scalpel at a magnification of × 100 from hematoxylin–eosin (H&E) stained slides. The suitability of the material was evaluated by hematoxylin and eosin staining, and care was taken to select tumor areas. Total RNA isolation was performed by commercially available RNA extraction kits designed for paraffin material according to the manufacturer’s instructions (miRNeasy FFPE kit; Qiagen, Hilden, Germany).

RT reactions were performed using 10 ng total RNA in a volume of 15 μl with the following conditions: 16 °C for 30 min, 42 °C for 30 min, 85 °C for 5 min, and 4 °C for 5 min. Expression levels of all genes studied and internal reference were examined using a fluorescence-based real-time detection method (ABI PRISM 7900 Sequence Detection System—Taqman; Applied Biosystems, Foster City, CA,). The following TaqMan gene expression assays (Applied Biosystems) were used according to the manufacturer’s instructions: *ASCL1* (HS00269932_m1), *DLL3* (HS01085096_m1), *INSM1* (Hs00357871_s1), *MYCL1* (Hs00420495_m1), *NEUROD1* (HS01922995_s1), *NOTCH1* (Hs01062014_m1), *POU2F3* (Hs00205009_m1), *YAP1* (Hs00902712_g1), *ACTB* (Hs01060665_g1) assay served as housekeeping reference gene for the analyses.

Each measurement was performed in duplicate. The ΔΔCt values were calculated subtracting ΔCt values of sample and ΔCt value of Stratagene (a pool of RNA derived from normal different tissues; Stratagene, CA), and converted to ratio by the following formula: 2 − ΔΔCt.

### Statistical analysis

Statistical analyses were carried out using the Stata 15.0 software (StataCorp, College Station, TX, U.S.A.). The differences in the distribution of the variables evaluated based on clinical-pathological parameters were analyzed using parametric and non-parametric tests (Student’s *t* test, Pearson’s chi-square test and Bonferroni’s correction, Wilcoxon’s rank test).

Time to relapse (disease-free interval, DFI) was assessed from the date of diagnosis to the date of relapse or the date of the last checkup. All dead patients were considered as events. Survival analysis was determined by the Kaplan–Meier curves and Mantel log-rank test was used to compare statistical differences. Cox regression analyses were carried out on DFI to calculate HRs and 95% CIs for the different study groups. All statistical tests were two sided. *p*-values < 0.05 were considered significant.

## Results

### Clinico-pathological characteristics of the study group

The clinical and pathological features of 46 neuroblastoma cases are summarized in Supplementary Table 1. In brief, the case series was composed of 20 females (43.5%) and 26 male patients (56.5%), 27 of them aged < 18 months (58.7%) and 19 patients ≥ 18 months (41.3%). Stages I–II, III, IV, and IVS were diagnosed in 15 (32.6%), 5 (10.9%), 18 (39.1%), and 8 (17.4%) cases, respectively. The poorly differentiated subtype (according to INPC) was present in the majority of cases (26/46, 56.5%), while undifferentiated and differentiated forms were seen in 17/46 (37%) and 3/46 (6.5%) cases, respectively. Low, intermediate, and high MKI were present in 21 (45.7%), 20 (43.5%), and 5 (10.8%) cases, respectively. Both calcifications and necrosis were noted in 28/46 cases (60.9%). Relapse occurred in 19/46 (41.3%) patients and 8/46 died of disease (17.4%).

### Gene expression profiles and correlations with clinical-pathological features

A strong reciprocal positive correlation was observed across the series between *NOTCH1* and *ASCL1* (*R* = 0.4561; *p* = 0.0014), *NEUROD1* (*R* = 0.4982; *p* = 0.0004), *INSM1* (*R* = 0.4763; *p* = 0.0008), and *YAP1* (*R* = 0.8475; *p* < 0.0001). Moreover, *ASCL1* was found significantly correlated with *DLL3* (*R* = 0.3685; *p* = 0.0117), *NEUROD1* (*R* = 0.3976; *p* = 0.0062) and *INSM1* (*R* = 0.5996; *p* < 0.0001). Furthermore, *NEUROD1* was significantly correlated with *MYCL1* (*R* =  − 0.2968; *p* = 0.0452) and *YAP1* (*R* = 0.5290; *p* = 0.0002), while *INSM1* resulted significantly correlated with *YAP1* (*p* = 0.0257).

As to concern clinical and pathological correlations (Supplementary Table 2), *NOTCH1*, *NEUROD1*, *MYCL1*, and *YAP1* were found significantly correlated with tumor stage. In detail, *NOTCH1* (*p* = 0.005), *NEUROD1* (*p* = 0.0059), and *YAP1* (*p* = 0.0008) were more expressed in stage IV tumors, while highest levels of *MYCL1* and *ASCL1* were seen in stages IVS and III, respectively (*p* = 0.0182 and *p* = 0.0134). Moreover, a higher level of both *NOTCH1* (*p* = 0.0079) and *YAP1* (*p* = 0.0026) was found in cases with differentiating morphology according to INPC classification. Finally, cases with high MKI according to Shimada demonstrated significantly lower levels of *POU2F3* (*p* = 0.0277).

### Gene expression levels and survival

**Fig. 1:**
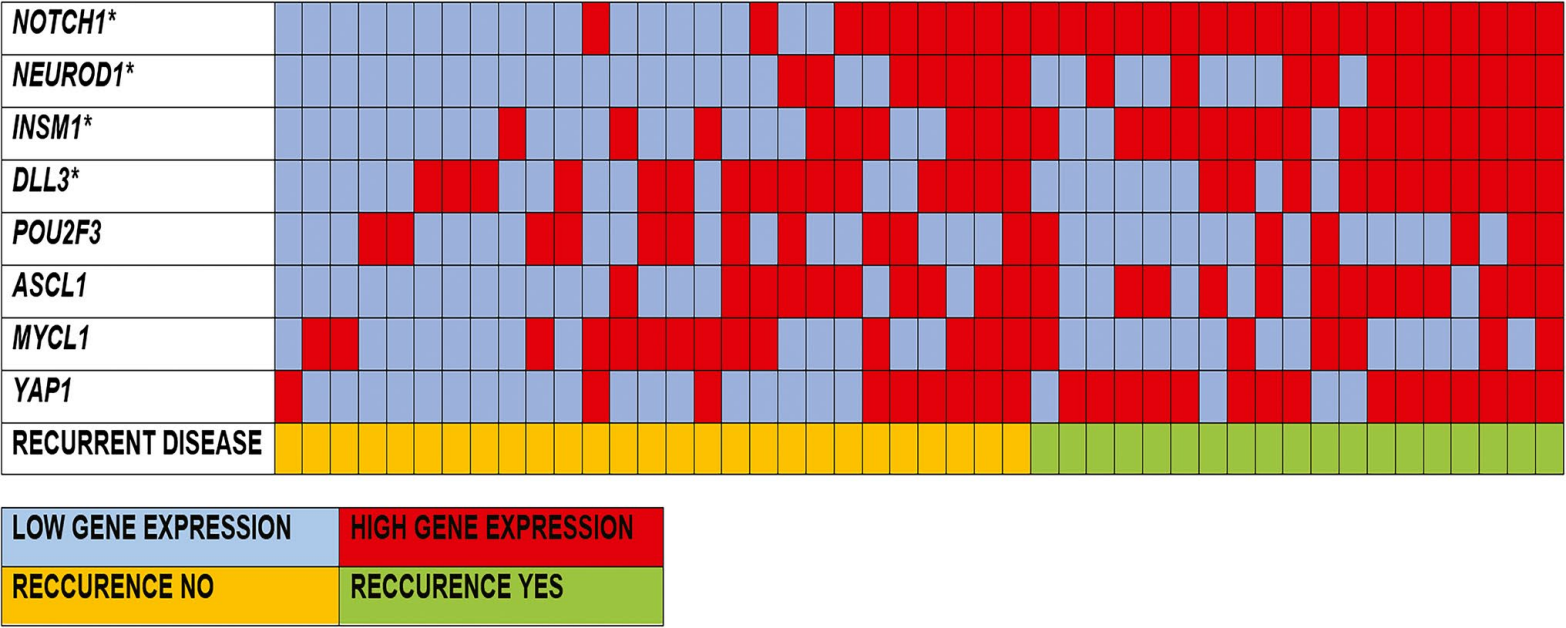
Gene expression patterns of the entire cohort according to the recurrent disease status. *Fisher exact test: NOTCH1: *p* < 0.001, NEUROD1: *p* = 0.037, INSM1: *p* = 0.001, YAP1: *p* = 0.003

In the overall series, cases with a high gene expression level (as determined using the median value) of *NOTCH1*, *INSM1*, *NEUROD1*, and *YAP1* demonstrated more frequently recurrence of the disease than those with low expression (Fig. 1). Considering the 30 treatment-naïve specimens only, we observed significantly higher gene expression levels of *ASCL1* (*p* = 0.0007), *INSM1* (*p* = 0.0016), and *DLL3* (*p* = 0.0064) in cases that relapsed during the follow-up (Table 2).

**Table 2:** Gene expression levels according to the follow up status in 30 treatment-naïve cases

Mean ± SD value	Total	Disease-free (#25)	Relapse (#5)	*P* value
*YAP1*	2.70 ± 3.11	2.67 ± 3.34	2.88 ± 1.21	0.891
*MYCL*	186.2 ± 229.6	231.7 ± 240.6	48.7 ± 81.8	0.145
*ASCL1*	379.2 ± 662.61	208.8 ± 378.7	1231.2 ± 110.7	0.0007
*POU2F3*	0.27 ± 0.58	0.29 ± 0.62	0.19 ± 0.37	0.744
*DLL3*	20.4 ± 49.1	9.90 ± 23.3	72.0 ± 100.4	0.0064
*INSM1*	2862.1 ± 5349.5	1565.4 ± 2369.1	9345.1 ± 10,523.1	0.0016
*NEUROD1*	0.16 ± 0.46	0.10 ± 0.28	0.49 ± 0.95	0.08
*NOTCH1*	1.62 ± 1.44	1.53 ± 1.51	2.06 ± 1.01	0.456

**Fig. 2:**
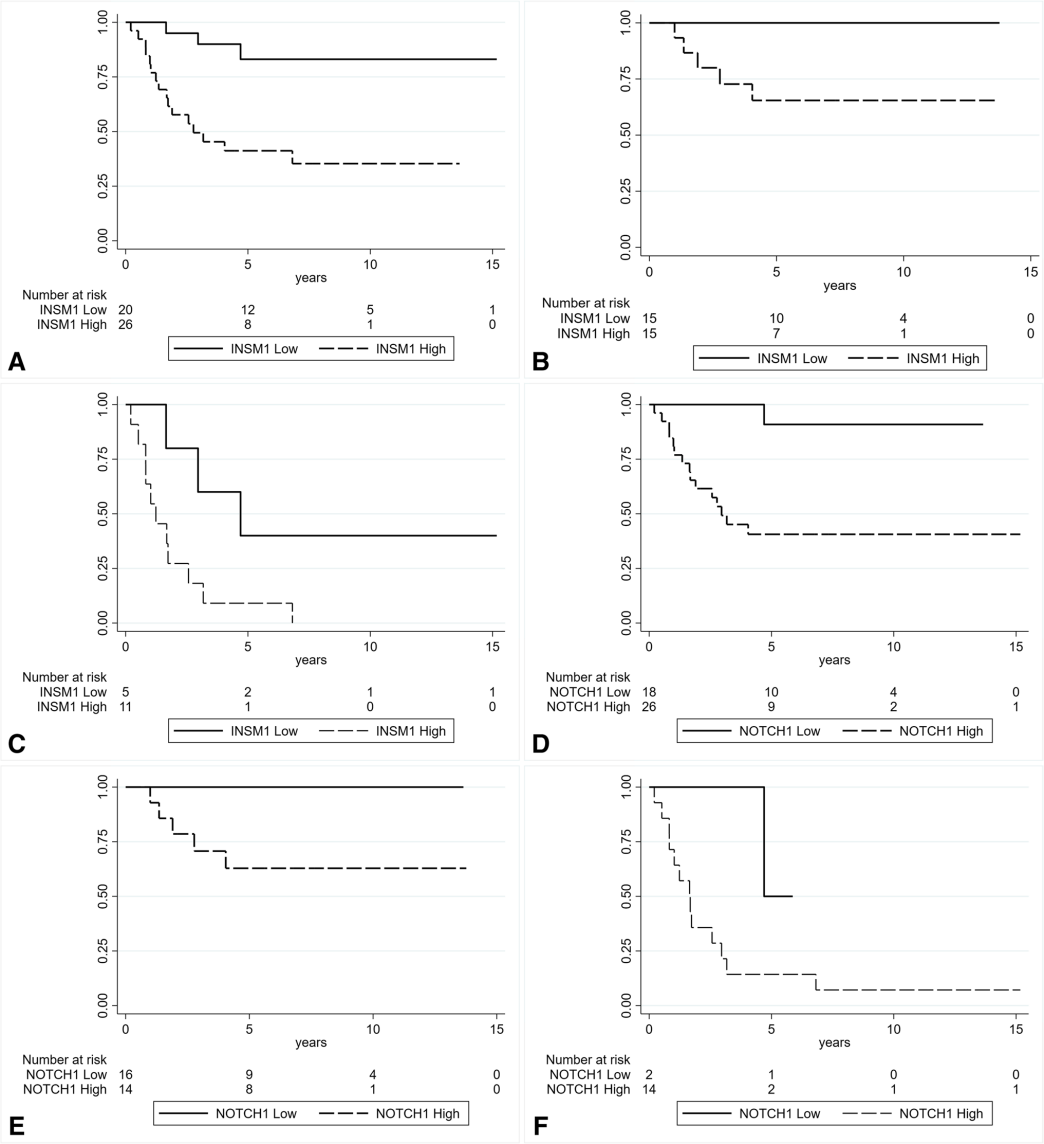
Kaplan–Meier estimates of DFI according to the *INSM1* gene expression level in the whole series (**A** *p* = 0.0012) and in treatment naïve (**B** *p* = 0.0147) and post-chemotherapy cases (**C** *p* = 0.0365). Kaplan–Meier estimates of DFI according to the *NOTCH1* gene expression level in the whole series (**D** *p* = 0.0001) and in treatment naïve (**E** *p* = 0.0085) and post-chemotherapy cases (**F** *p* = 0.1580)

As to concern univariate survival analyses, higher levels of *INSM1* were significantly correlated with shorter disease-free interval (DFI) in the whole group (Fig. 2A, *p* = 0.0012), as well as when separately analyzed in the treatment-naïve (Fig. 2B, *p* = 0.0147) and the post-chemotherapy groups of patients (Fig. 2C, *p* = 0.0365). Moreover, the patients with higher levels of *NOTCH1* had a shorter DFI, both in the whole cohort (Fig. 2D, *p* = 0.0001) and in the 30 treatment naïve patients (Fig. 2E, *p* = 0.0085), (but not in the 16 post-chemotherapy patients, Fig. 2F, *p* = 0.158). In addition, considering the whole case series, patients with high expression of *YAP1* and *NEUROD1* had shorter DFI, compared to those with low expression (Supplementary Fig. 1: A, *p* = 0.0007 and B, *p* = 0.0128). However, no significance was observed when patients were stratified according to the chemotherapy status (data not shown).

Furthermore, Cox regression analyses to estimate HRs and 95% CIs for DFI in the different study groups shown in Table 3 demonstrated in the whole series that stage IV (HR 8.57; CI 1.1–68.6 *p* = 0.043) and high expression of *NOTCH1* (HR 15.6; CI 2.05–118.7, *p* = 0.008), *NEUROD1* (HR 3.17;1.14–8.78, *p* = 0.026), *INSM1* (HR 5.24, CI 1.49–18.5, *p* = 0.010), and *YAP1* (HR 8.57, CI 1.93–37.9, *p* = 0.005) were associated with adverse prognosis. Moreover, poorly differentiated forms (HR 0.24; 0.06–0.98, *p* = 0.048) of disease were significantly associated to better DFI survival compared to undifferentiating lesions.

**Table 3:** DFI Univariate survival analysis whole series (#46)

Parameter		HR [CI]	*p*
Age	(< 18 vs ≥ 18 months)	3.97 [0.0–inf]	1.000
Stage	I/II	1	
	III	5.16 [0.0–inf]	1.000
	IV	8.57 [1.1–68.6]	0.043
	IVS	4.91 [0.0–inf]	1.000
INPC	Undifferentiated	1	
	Poorly differentiated	0.24 [0.06–0.98]	0.048
	Differentiated	0.52 [0.06–4.47]	0.549
Shimada	Low MKI*	1	
	Intermediate MKI	1.90 [0.54–6.65]	0.315
	High MKI	8.24 [- -]	-
*NOTCH1*	Low vs high	15.6 [2.05–118.7]	0.008
*ASCL1*	Low vs high	2.09 [0.76–5.78]	0.153
*DLL3*	Low vs high	1.26 [0.46–3.49]	0.649
*NEUROD1*	Low vs high	3.17 [1.14–8.78]	0.026
*INSM1*	Low vs high	5.24 [1.49–18.5]	0.010
*YAP1*	Low vs high	8.57 [1.93–37.9]	0.005
*POU2F3*	Low vs high	0.61 [0.16–2.32]	0.473
*MYCL1*	Low vs high	0.34 [0.11–1.08]	0.069

Abbreviations: *MKI, mitosis-karyorrhexis index; *HR*, hazard ration; *CI*, confidence intervals

### Gene combination analyses

Considering the different combinations of *INSM1*, *NOTCH1*, *NEUROD1*, and *YAP1* gene expression levels according to the recurrent disease status (Supplementary Table 3), patients with low expression of all four genes did not experience recurrent disease while patients with high levels of at least one of the aforementioned genes demonstrated significantly more frequent recurrences (*p* = 0.003).

Moreover, we observed a statistically significant increase in the odds of the patients with the high levels of ≥ 3 above indicated genes when considering the whole case series (*OR* = 8.75, 95% *CI* = 2.19–34.81, *p* = 0.002). However, when stratified according to the treatment status, no significance was observed the treatment naïve group (*OR* = 4.75, 95% *CI* = 0.63–35.48, *p* = 0.129) and post-chemotherapy group of patients (*OR* = 2.75, 95% *CI* = 0.16–46.79, *p* = 0.484) analyzed separately (Supplementary Table 4).

## Discussion

We demonstrated in a series of 46 pediatric neuroblastomas that neuroendocrine-lineage transcriptional genes are expressed in neuroblastoma and that their profiles of expression may identify subgroups of patients with increased risk of recurrence and/or shorter survival. The study design focused on gene expression analysis and not immunohistochemical determination of the corresponding protein expression due to the following considerations. First, reliable and robust antibodies are not available for all molecules. Second, tissue material available was limited in some cases preventing the possibility to perform both RNA extraction and subsequent sectioning for a multi-target immunohistochemical procedure. Moreover, even in the more extensively investigated small cell lung cancer, model gene expression data have been used for molecular classification purposes [11, 12], whereas no study clearly demonstrated a linear correlation between protein and mRNA expression for these targets. The strength of the transcriptional data observed in this study is proven by the very high positive reciprocal correlation among most of the markers investigated, being *NOTCH1* the one showing the correlation with the highest number of genes. Interestingly, at variance with other models in which these transcription factors are active, such as small cell lung carcinoma, the expression of the genes here investigated did not segregate neuroblastoma cases into different families characterized by alterative transcriptional profiles, since no inverse correlation among any of the genes tested was identified.

Our data on the association of gene expression profiles with aggressive clinical outcome and survival represent the first global evidence that the expression of neuroendocrine differentiation transcriptional drivers may be used to further characterize neuroblastoma patients, and several preclinical evidence exist supporting their role as potentially relevant clinical prognostic biomarkers.

Among all genes, four of them—*NOTCH1*, *INSM1*, *YAP1*, and *NEUROD1*—emerge as the most significant, being associated with both shorter disease-free interval and high tumor stage and/or risk of recurrence.

The association of *NOTCH1* overexpression with adverse clinical outcome in our series is expanding previous data obtained by means of the immunohistochemical analysis of Notch1 protein in a large series of neuroblastomas [33]. At variance with such previous series, we did not compare the expression of *NOTCH1* (and of all other genes) with *MYCN* amplification status, and this limitation should be considered in future studies. Interestingly, in a translational view, the demonstration of *NOTCH1* expression in neuroblastoma and its positive correlation with aggressiveness is paralleled by in vitro data on the efficacy of Notch1 inhibition in neuroblastoma cells. In a study on a variety of neuroblastoma cell lines, gamma-secretase inhibition (in particular GSI-I) was shown to impair cell proliferation and to promote apoptosis in vitro and in vivo through targeting Notch signaling [34]. Moreover, the Notch1 inhibitor NSI-1 has recently shown to suppress the viability of SH-SY5Y neuroblastoma cells characterized by a constitutive Notch1 activation [35]. The impact of *NOTCH1* overexpression in the adverse clinical behavior of neuroblastoma patients in our series is to be further validated in a biological perspective. However, among the possible mechanisms, Notch1 has been shown to actively maintain a stem cell phenotype in neuroblastoma cells that confer highly tumorigenic properties [36].

*INSM1* transcription factor has emerged in vitro as a neuroblastoma biomarker that plays critical role in facilitating tumor cell growth and transformation [30]. Its protein nuclear expression has been documented in 84% of neuroblastomas, with a suggested association with clinical outcome, being the three INSM1-negative neuroblastoma patients in the published study all alive with a median survival of 15 years as opposed to a median of 5 years in 9 out of 13 INSM1-positive neuroblastoma patients [28]. No definitive data are present in the literature on its possible role as prognostic biomarker in neuroblastoma. However, in other tumor models—such as pulmonary high-grade neuroendocrine carcinoma-positive INSM1 protein expression has been associated with a dismal prognosis [37], in line with the data on *INSM1* gene expression levels from the present study.

Among other markers associated with neuroblastoma aggressiveness in our series, *NEUROD1* was already shown to promote cell growth in vitro [24] and tumor formation in vivo [38] in neuroblastoma cells. YAP1 expression has been reported to significantly increase cell proliferation and growth through inhibition of 27^Kip1^ activity in neuroblastoma cell lines SH-SY5Y and SK-N-SH [39]. Moreover, YAP1 overexpression has been associated to the increased resistance to platinum-based [40] and MEK-inhibiting [41] therapeutic strategies.

In terms of correlation with differentiation, *NOTCH1* and *YAP1* expression was significantly higher in differentiating compared to poorly differentiated tumors according to the INPC classification. This observation cannot find a clear explanation in the current literature. A previous in vitro study is partly in contrast with our findings and shows that Notch1 inhibition prevents neurite formation in neuroblastoma cells [42]. Moreover, these data are apparently in contrast with the adverse impact on clinical outcome for both markers in our same series and might more probably represent a bias related to the limited number of cases in the differentiation group.

The potential clinical impact of our findings is twofold. First, the integration of gene expression profiles of the biomarkers investigated in the present study might assist to better predict the clinical behavior of neuroblastoma patients, and to improve a personalized approach in tailoring adjuvant chemotherapeutic regimens and/or predict response to treatment. Second, the pathways regulated by the genes herein investigated are potential targets for selective therapies. In particular, DLL3 has been tested as a target for rovalpituzumab tesirine therapy in neuroblastoma models with promising results [43].

In conclusion, we identify a strong prognostic impact of neuroendocrine-lineage transcriptional profiles in neuroblastoma, and suggest that the evaluation of *NOTCH1*, *INSM1*, *YAP1*, and *NEUROD1* might help to further characterize the risk of relapse in neuroblastoma patients.

## Supplementary Information

Below is the link to the electronic supplementary material.

Supplementary file 1 (DOCX 26 kb)

Supplementary file 2 (PNG 469 kb)

High resolution image (TIF 19566 kb)
