# Supplementary material for: Overexpression of INSM1, NOTCH1, NEUROD1, and YAP1 genes is associated with adverse clinical outcome in pediatric neuroblastoma
Source: Virchows Arch. 2022 Sep 19;481(6):925–33. doi: 10.1007/s00428-022-03406-4 (PMC9734219; doi:10.1007/s00428-022-03406-4)
Supplement: Supplementary file 1 — (DOCX 26 kb) [file 428_2022_3406_MOESM1_ESM.docx]

**Supplementary Table 1.** Clinico-pathological characteristics of a 46 neuroblastoma case series.

|  |  | Total #46 |
| --- | --- | --- |
| Sex | F | 20 (43.5%) |
|  | M | 26 (56.5%) |
| Age | <18 months | 27 (58.7%) |
|  | ≥ 18 months | 19 (41.3%) |
| Treatment | Treatment-naive | 30 (65.2%) |
|  | Post-treatment | 16 (34.8%) |
| Site | Adrenal gland | 38 (82.6%) |
|  | Other* | 8 (17.4%) |
| Stage | I/II | 15 (32.6%) |
|  | III | 5 (10.9%) |
|  | IV | 18 (39.1%) |
|  | IVS | 8 (17.4%) |
| INPC classification | Undifferentiated | 17 (37%) |
|  | Poorly differentiated | 26 (56.5%) |
|  | Differentiated | 3 (6.5%) |
| Shimada  classification | Low MKI° | 21 (45.7%) |
|  | Intermediate MKI | 20 (43.5%) |
|  | High MKI | 5 (10.8%) |
| Calcifications | No | 18 (39.1%) |
|  | Yes | 28 (60.9%) |
| Necrosis | No | 18 (39.1%) |
|  | Yes | 28 (60.9%) |
| Relapse | No | 27 (58.7%) |
|  | Yes | 19 (41.3%) |
| Death | No | 38 (82.6%) |
|  | Yes | 8 (17.4%) |

*Mediastinal, pelvic, and cervical localization; °MKI: mitosis-karyorrhexis index.

**Supplementary Table 2.** Correlation of gene expression levels with major clinical and pathological variables (*MKI: mitosis-karyorrhexis index).

|  |  | ***NOTCH1*** | ***ASCL1*** | ***DLL3*** | ***NEUROD1*** | ***INSM1*** | ***POU2F3*** | ***MYCL1*** | ***YAP1*** |
| --- | --- | --- | --- | --- | --- | --- | --- | --- | --- |
| **Age** | <18 months | 1.11 | 103.2 | 1.25 | 0.001 | 998.7 | 0.03 | 103.2 | 1.70 |
|  | ≥18 months | 2.39 | 74.4 | 2.13 | 0.01 | 2771.2 | 0.03 | 74.4 | 2.93 |
|  | *P* value | *0.1901* | *0.6426* | *0.6586* | *0.1682* | *0.5214* | *0.6113* | *0.2874* | *0.1547* |
| **Stage** | I/II | 0.96 | 25.6 | 0.15 | 0.0002 | 789.5 | 0.01 | 129.3 | 1.43 |
|  | III | 1.11 | 604.3 | 15.9 | 0.01 | 843.9 | 0.39 | 37.5 | 1.60 |
|  | IV | 3.56 | 203.8 | 3.80 | 0.065 | 4845.8 | 0.03 | 56.1 | 6.48 |
|  | IVS | 1.41 | 87.5 | 3.01 | 0.002 | 731.4 | 0.10 | 253.5 | 3.12 |
|  | *P* value | *0.005* | *0.0134* | *0.4071* | *0.0059* | *0.1145* | *0.3413* | *0.0182* | *0.0008* |
| **INPC** | Undifferentiated | 2.89 | 389.9 | 4.09 | 0.03 | 3609.8 | 0.03 | 46.1 | 3.09 |
|  | Poorly differentiated | 1.08 | 55.4 | 0.39 | 0.001 | 753.3 | 0.03 | 105.9 | 1.44 |
|  | Differentiated | 3.52 | 134.4 | 8.91 | 0.149 | 907.4 | 0.02 | 9.63 | 14.7 |
|  | *P* value | *0.0079* | *0.0521* | *0.3671* | *0.1130* | *0.2048* | *0.7854* | *0.0607* | *0.0026* |
| **Shimada** | Low MKI* | 1.30 | 119.6 | 2.12 | 0.0006 | 998.8 | 0.02 | 129.3 | 1.79 |
|  | Intermedium MKI | 2.51 | 84.7 | 0.79 | 0.03 | 1174.3 | 0.10 | 76.3 | 2.98 |
|  | High MKI | 1.48 | 91.9 | 5.60 | 0.002 | 986.4 | 0.001 | 29.0 | 2.33 |
|  | *P* value | *0.3757* | *0.6618* | *0.7353* | *0.2160* | *0.6670* | *0.0277* | *0.1662* | *0.4839* |

**Supplementary Table 3.** Gene combination analyses *INSM1, NOTCH1, NEUROD1*, and *YAP1* according to recurrent disease status.

| **Recurrent disease** | **None^a^** | **1-gene- high levels^b^** | **2-genes- high levels^c^** | **3-genes- high levels^d^** | **4-genes- high levels^e^** | ***P* value** |
| --- | --- | --- | --- | --- | --- | --- |
| No | 11 | 6 | 3 | 5 | 3 | 0.003 |
| Yes | 0 | 1 | 3 | 5 | 9 |  |
| Total | 11 | 7 | 6 | 10 | 12 |  |

*Bonferroni correction e vs a p=0.001; e vs b p=0.038

**Supplementary Table 4.** OR and 95% CI regarding different gene expression levels of *INSM1, NOTCH, NEUROD1* and *YAP1.*

| Recurrent disease | OR | 95% CI | *P* value |
| --- | --- | --- | --- |
| Whole cohort #46 | | | |
| ≥ 3 markers with high gene expression | 8.75 | 2.19-34.81 | 0.002 |
| 30 treatment naïve | | | |
| ≥ 3 markers with high gene expression | 4.75 | 0.63-35.48 | 0.129 |
| 16 post-chemotherapy | | | |
| ≥ 3 markers with high gene expression | 2.75 | 0.16-46.79 | 0.484 |

OR: Odds ratio; CI: Confidence Interval.
