# Supplementary figures and images for: Overexpression of INSM1, NOTCH1, NEUROD1, and YAP1 genes is associated with adverse clinical outcome in pediatric neuroblastoma
Source: Virchows Arch. 2022 Sep 19;481(6):925–33. doi: 10.1007/s00428-022-03406-4 (PMC9734219; doi:10.1007/s00428-022-03406-4)

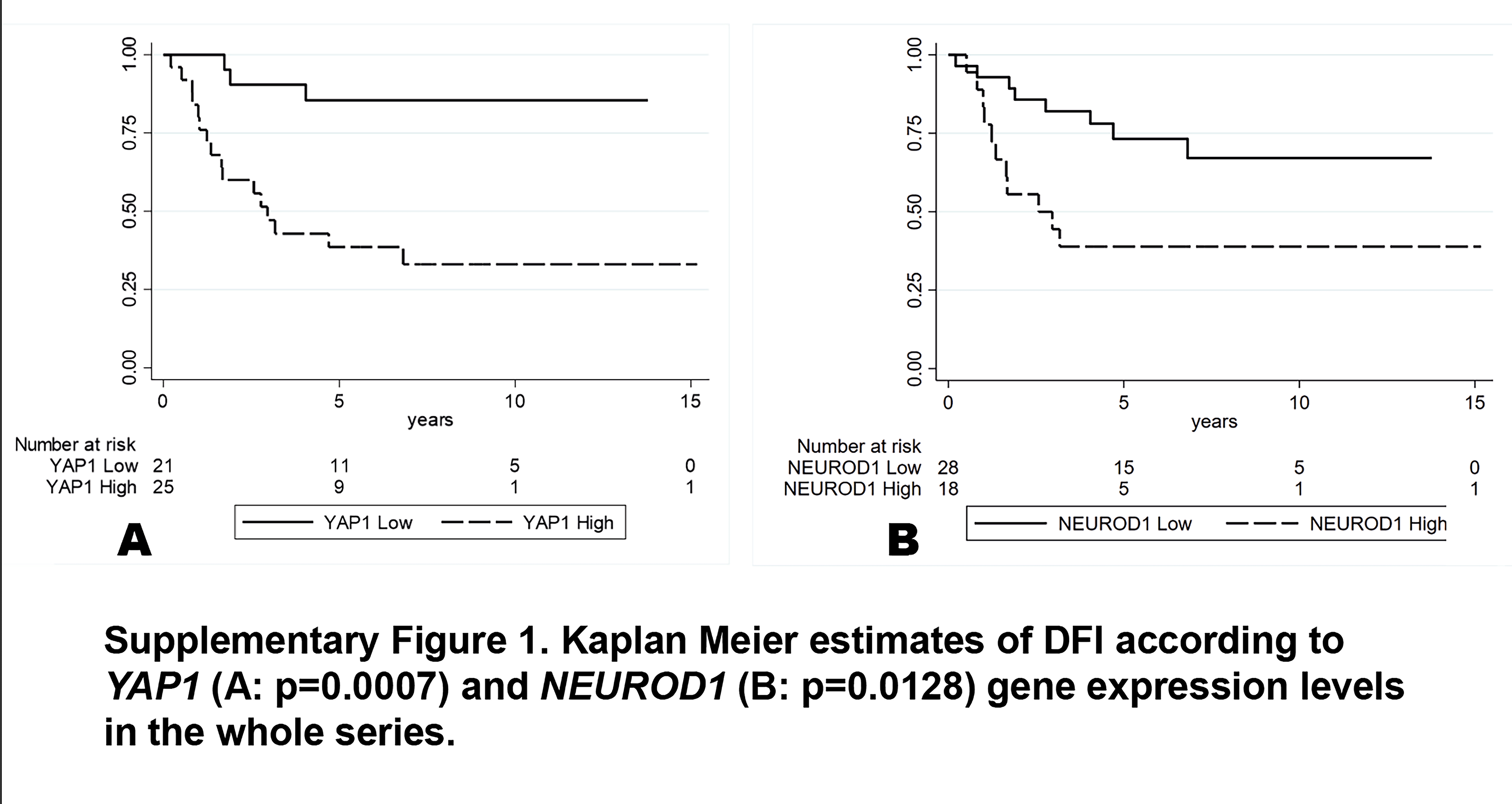

Supplement: Supplementary file 2 — (PNG 469 kb) [file 428_2022_3406_Fig3_ESM.png]

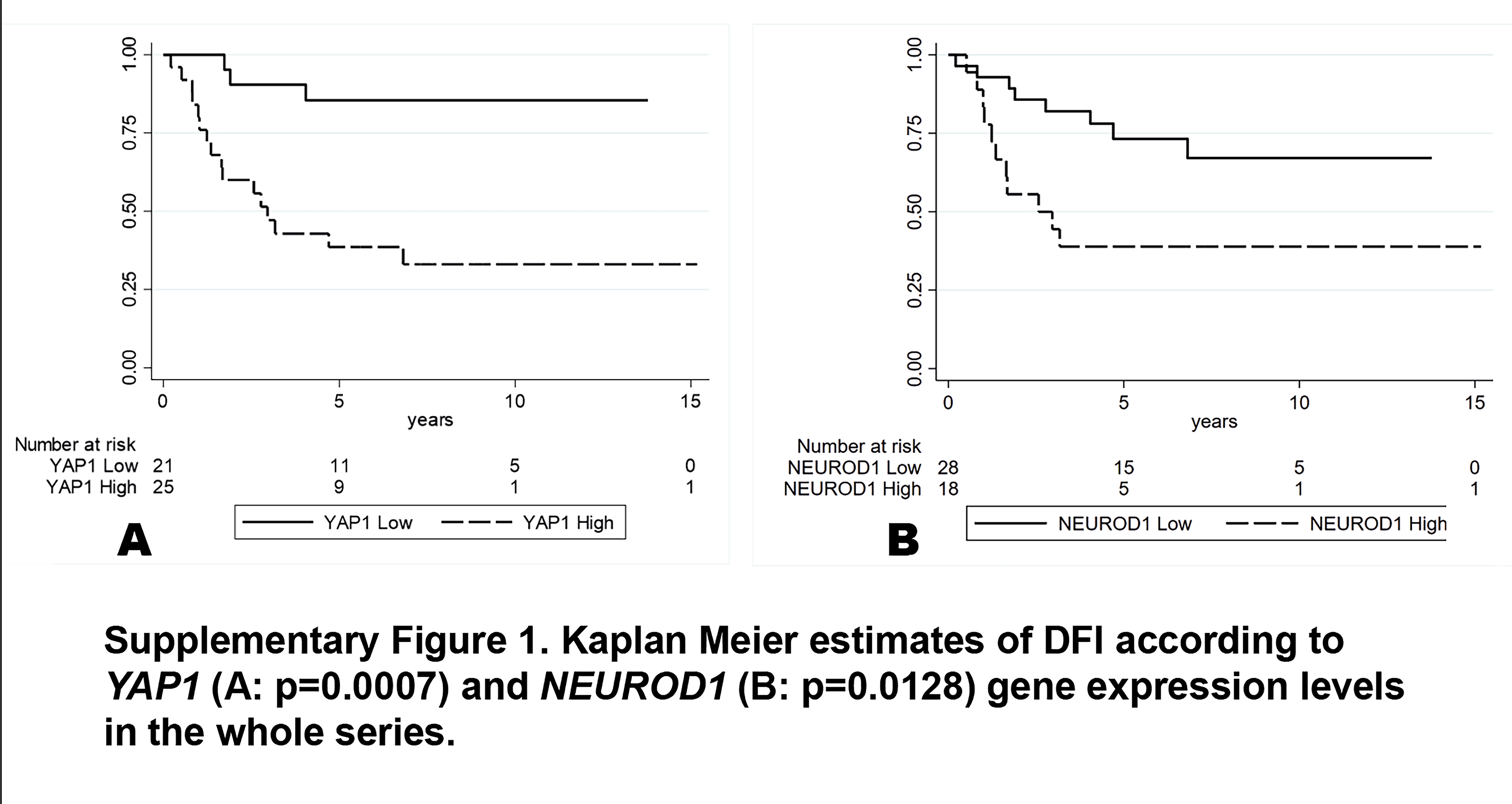

Supplement: Supplementary file 3 — High resolution image (TIF 19566 kb) [file 428_2022_3406_MOESM2_ESM.tif]
